# Biogeochemical impacts of flooding discharge with high suspended sediment on coastal seas: a modeling study for a microtidal open bay

**DOI:** 10.1038/s41598-021-00633-8

**Published:** 2021-11-04

**Authors:** Yasuhiro Hoshiba, Hiroyasu Hasumi, Sachihiko Itoh, Yoshimasa Matsumura, Satoshi Nakada

**Affiliations:** 1 Atmosphere and Ocean Research Institute, The University of Tokyo, 5-1-5, Kashiwanoha, Kashiwa, Chiba 277-8564 Japan; 2 National Institute for Environmental Studies, 16-2, Onogawa, Tsukuba, Ibaraki 305-8506 Japan

**Keywords:** Biogeochemistry, Environmental sciences, Natural hazards, Ocean sciences

## Abstract

Freshwater, suspended sediment matter (SSM), and nutrients discharged from rivers into the ocean have large impacts on biological production. In particular, during floods, coastal areas are greatly stirred up and large amounts of nutrients are supplied to the sea surface. We investigate the biogeochemical impact of flooding river discharges containing a large amount of SSM by conducting numerical simulations for a specific flooding event of the Yura River, Japan. Parameters are varied over wide ranges of SSM properties and nutrient content in riverine water. Two qualitatively different regimes of the riverine plume, hypopycnal and hyperpycnal, appear within realistic parameter ranges. Compared with the reference case without SSM, the surface salinity (nutrients) within the riverine plume becomes lower (higher) in hypopycnal cases and higher (lower) in hyperpycnal cases within a few days after the flooding discharge. These results suggest the necessity of properly taking into account the effect of SSM in assessing the influence of high river discharges on coastal biogeochemistry. It is the case not only for the specific river and event we are dealing with but also for other flooding events and other rivers and connecting coastal seas.

## Introduction

Riverine water is one of the most dominant factors controlling the circulation patterns in coastal seas. Discharged riverine water typically spreads horizontally in a thin surface layer by forming a plume of low-salinity water^1^; in addition, it induces vertical estuarine circulation, in which offshore export of a water mass near the surface is compensated for by onshore inflow beneath^2^. The behavior of riverine water in coastal seas depends on the difference in density between riverine water and ambient seawater.

**Figure 1:**
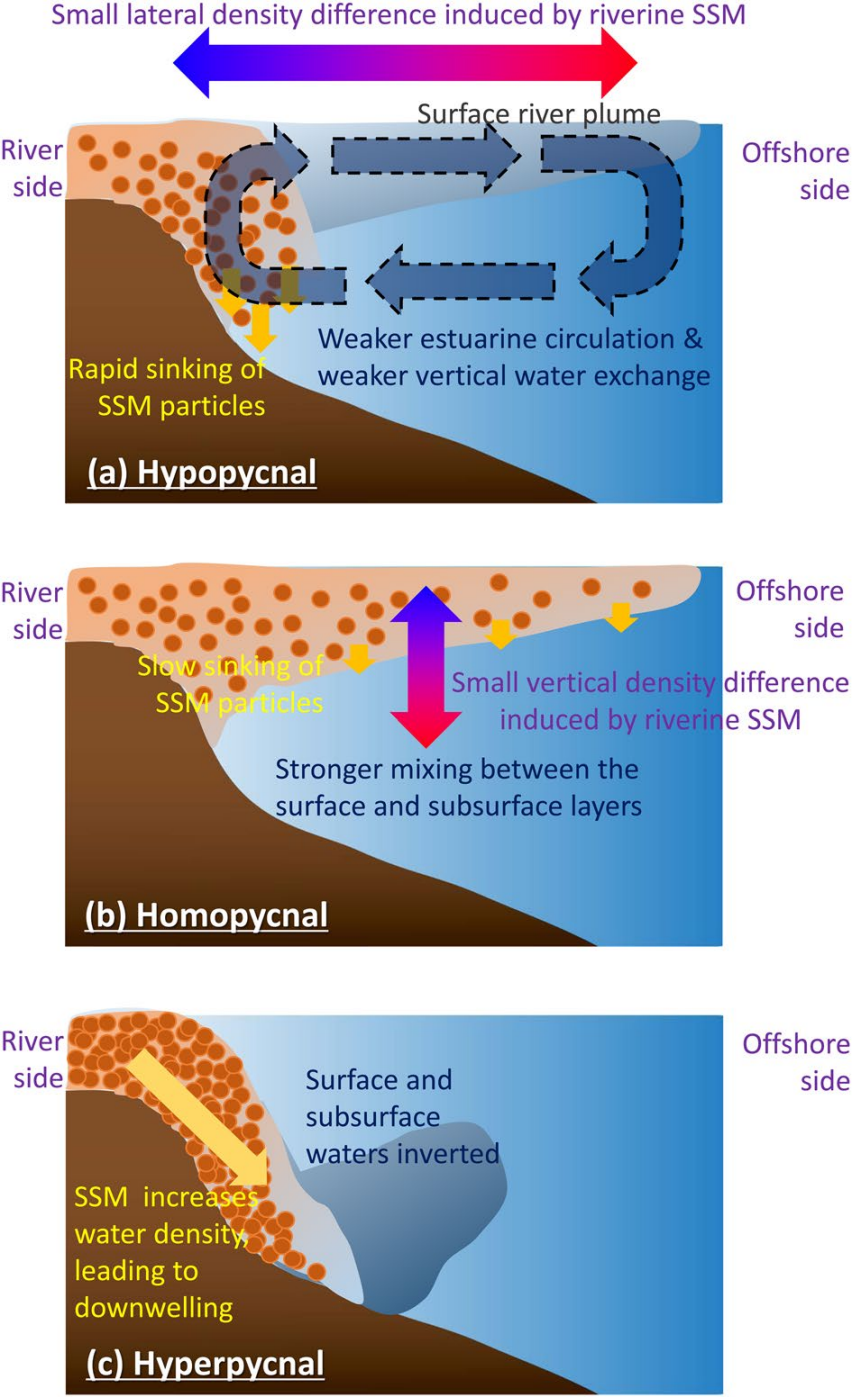
Schematic representation of processes of suspended sediment matter (SSM) and physical field interaction in (**a**) hypopycnal plume, (**b**) homopycnal plume, and (**c**) hyperpycnal plume cases. Figures were created using Adobe Illustrator CC 2018 and Microsoft Powerpoint 2013.

The density of riverine water is influenced by salinity and temperature, and suspended sediment matter (SSM) also has a marked effect in some circumstances (typically, but not exclusively, flood events). River plumes affected by SSM are classified into three categories^3^, namely hypopycnal, homopycnal, and hyperpycnal. Hypopycnal mode, the most common plume state, occurs when the density of riverine water including SSM is lower than that of seawater, and the riverine plume basically moves in a thin surface layer. A series of mechanisms by which SSM affects hypopycnal plumes has been demonstrated^4^: (1) the horizontal density difference between the riverine and offshore waters is reduced by the discharged SSM content near the river mouth; (2) the estuarine circulation is weakened due to the reduced density difference; and (3) the weakened vertical estuarine circulation suppresses vertical water exchange between the surface and sub-surface layers (Fig. 1a). Hypopycnal mode is likely to occur when SSM has a shorter residence time in the water column (i.e., relatively large particle size and low abundance). In contrast, if SSM has a relatively long residence time (i.e., relatively small particle size and high abundance), homopycnal-mode processes^5^ work as follows: (1) SSM spreads over a larger area due to the smaller particle sinking rate; (2) the vertical density difference between the surface and sub-surface layers is reduced as a consequence of the higher density in the whole surface layer resulting from residual SSM; and (3) the salinity in the surface layer increases owing to enhanced vertical mixing (Fig. 1b). In the case of extremely high SSM concentration, the density of riverine water exceeds that of ambient seawater, resulting in a hyperpycnal plume in which river runoff sinks to the seafloor^6^ (Fig. 1c). In the present study, we explicitly consider hypopycnal and hyperpycnal modes. These two modes are distinguished by whether surface riverine water is isolated or mixed with sub-surface water. Homopycnal plumes are herein included in hyperpycnal mode, as such plumes mix water from upper and lower layers.

Large discharges of riverine water are known to induce phytoplankton blooms^7^, with direct nutrient supply from rivers often being the main source of such blooms^8^. In addition, river runoff drives river plumes and estuary circulation that are closely related to phytoplankton blooms^9^. The supply of nutrients to the surface water, which is a prerequisite for blooming, is derived from nutrients contained in the discharged water and from the subsurface as a consequence of vertical motion and associated mixing induced by the riverine water discharge^10^. The variations in nutrient supplies caused by the effects of mixing, entrainment, and advection by rivers have an influence on coastal primary production^11, 12^. SSM–physical interactions through the hypopycnal and hyperpycnal processes can substantially change nutrient-supply environments; however, to the best of the authors’ knowledge, no previous study has investigated how riverine water containing SSM affects the supply of nutrients to the sea surface.

**Figure 2:**
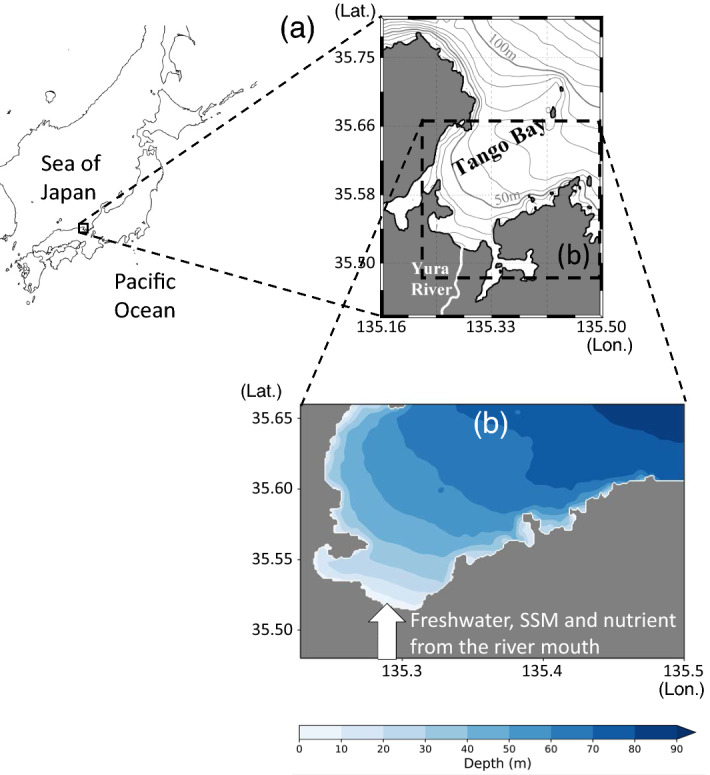
(**a**) Location and topography of Tango Bay and the Yura River, Japan. (**b**) Topography of the model domain, although a few small bays connected to the Tango Bay are treated as land-grids. We consider freshwater, SSM, and nutrients (nitrate) flowing from the southern river mouth into the sea (white arrow). This figure was prepared with matplotlib ver. 3.3.1 in Python ver. 3.7.6 and Microsoft Powerpoint 2013.

In this study, we investigate the influences of a large discharge containing a large amount of SSM from a flooding river on the nutrient supply to the sea surface in a coastal sea. For this purpose, we conduct numerical simulations of an actual flooding event of the Yura River, Japan, which flows through Tango Bay to the Japan Sea (Fig. 2). The flood caused by Typhoon “Man-yi” in September 2013 was the largest ever recorded in the Yura River discharge. The flood is expected to have a significant impact on this area since past high river discharges have enhanced primary production in the Tango Bay^13^ which provides important nursery grounds for many fish species^14, 15^. However, it is difficult to conduct spatially and temporally fine monitoring during flood events because massive sediment discharge would bury observational instruments, and resolutions tend to be insufficient for satellite observations. A modeling study would help illustrate the influences. As there are large uncertainties in the properties of SSM and the nutrient levels of flooding situations, we investigate the sensitivity to these factors over broad (but plausible in natural systems) ranges of parameters (Table 1). We quantitatively evaluate the effects of SSM–physical interaction for a range of scenarios, including hypopycnal to hyperpycnal situations, and consider the results in terms of the physical aspects and effect on the lower trophic ecosystem.

**Table 1:**
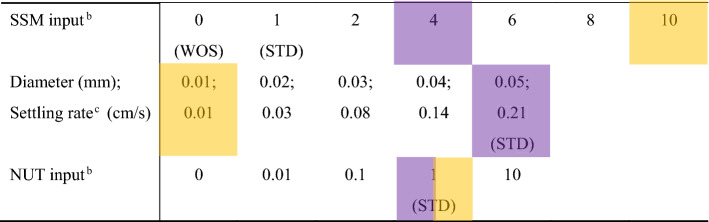
Parameters for the sensitivity experiments^a^.

Purple denotes the parameter combination in which the typical hypopycnal situation occurs; yellow indicates the parameters for the typical hyperpycnal situation.

^a^Three parameters are varied in each experiment except for the WOS (without SSM) case. WOS changes only with nutrient input, so there is a total of 155 experimental cases.

^b^Units of the inputs are normalized by the STD (Standard case) input. The input ranges of SSM and nutrients are determined on the basis of reasonable values for actual river systems^16, 17^.

^c^Settling speeds are estimated from Rubey (1933)^18^, using the diameters and composition (2650 kg/m^3^).

## Results

### River plume in the case without suspended sediment matter

**Figure 3:**
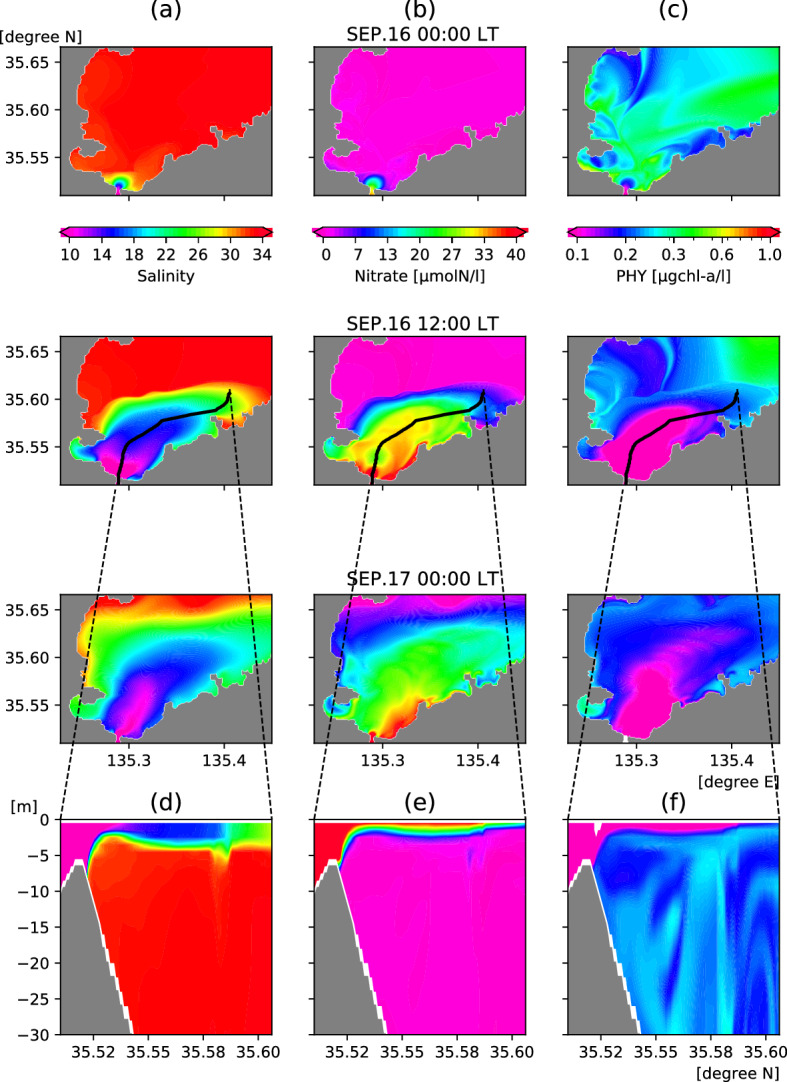
Surface (top two layers) mean distributions of (**a**) salinity, (**b**) nitrate, and (**c**) phytoplankton of WOS (the case without SSM). The time shifts from 00:00 local time (LT) on September 16 (top panels) to 00:00 LT on September 17 (third row of panels from the top), 2013. Bottom panels depict vertical (**d**) salinity, (**e**) nitrate, and (**f**) phytoplankton distributions along the center section of the river plume indicated by the black lines on the second row of panels. The black line marks the main streamline of the riverine output flows determined by the flow speed and the sea surface height maxima. The ecosystem model runs on a nitrogen basis. Unit conversion from nitrogen (μmolN/l) to chl.*a* (μg-chla/l) is the same way as OY05^28^. This figure was prepared with matplotlib ver. 3.3.1 in Python ver. 3.7.6.

In the case without SSM (WOS in Table 1), the freshwater discharge from the river causes only a hypopycnal plume. The largest flood in the river between 1953 and 2019 according to available observed data^19^ causes a large amount of low-salinity water to flow out of the river mouth, which spreads in an offshore direction with time (Fig. 3a). Owing to Earth's rotation, the low-salinity area (river plume) tends to spread more to the east. The plume area (salinity ≤ 28.0) reaches its maximum at around 00:00 local time (LT) on Sep. 17. Herein, we focus primarily on plume development under high riverine discharge prior to that time. The black line in the plume at 12:00 LT on Sep. 16 depicts the main flow axis calculated from the flow velocity and sea surface height. The vertical section of the flow axis (Fig. 3d) shows that the low-salinity water spreads above the surface layer (< 4 m depth). The presence of low-salinity water spreading thinly over the surface layer means that the plume is classified as a hypopycnal plume. In the absence of SSM, the plume generated by the targeted flood becomes a hypopycnal plume.

River-derived nitrate (Fig. 3b,e), similar to the standard case (STD in Table 1), exhibits a distribution of high and low concentrations that is the inverse of that for salinity. The nutrient concentration is larger inside the river plume and smaller outside. The concentration tendency of phytoplankton is temporarily opposite to that of nitrate and similar to that of salinity during plume development (Fig. 3c,f). This is because there is little marine phytoplankton (PHY) in river water, and the high concentration of PHY that existed initially near the river mouth is driven offshore by flooding. Such cases of PHY being transported offshore during floods, resulting in temporarily low concentrations, have been observed elsewhere^8, 20^.

We only deal with nitrate as the nutrient discharged from the river, but the mechanism of SSM-physical interaction discussed in this study applies also to other nutrients such as phosphate and silicate. Hence, we use the more general term “nutrients” instead of nitrate hereafter.

### Hypopycnal and hyperpycnal processes

**Figure 4:**
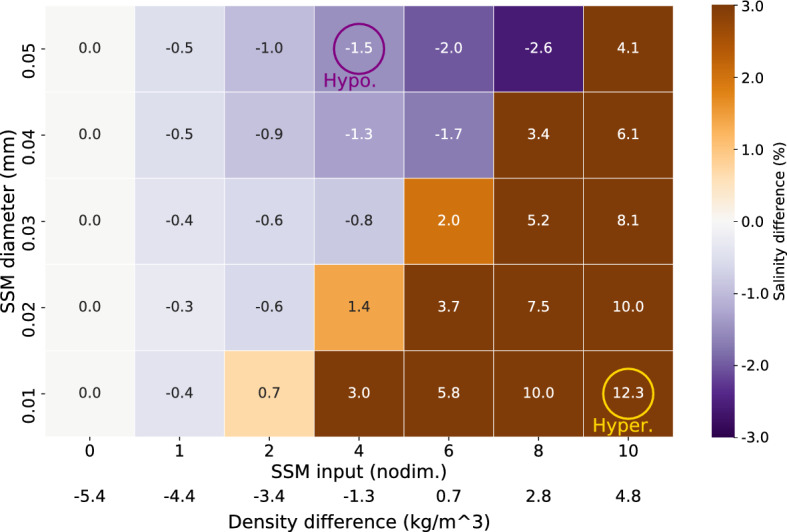
Mean salinity differences from WOS in the surface (the top two layers) river plume area (salinity ≤ 28.0), in sensitivity experiments with respect to changes in SSM input (x-axis) and diameter (y-axis), at the times of maximum differences. The SSM inputs of the x-axis are normalized by those of STD. Numbers on the lower x-axis are the density differences between river water and seawater, approximately calculated from the maximum amounts of input SSM and freshwater, temperature, and salinity in the initial state. Negative values (blue color) indicate lower salinity (i.e., a more intense hypopycnal plume) than WOS, whereas positive values (brown color) denote higher salinity (i.e., a hyperpycnal plume). The purple circle marks a typical hypopycnal case and the yellow circle a typical hyperpycnal one. *nodim*. non-dimensional.

When riverine SSM is introduced, the behavior of plumes changes in several ways. By changing the amount of SSM and the size of SSM particles (i.e., the rate of settling and removal from seawater) in the SSM–physical interaction, salinity changes can occur in the river plumes (Fig. 4). As explained in the Introduction, the processes are classified into two types: hypopycnal (surface lower-salinity) and hyperpycnal (surface higher-salinity) situations. In Fig. 4, a blue color suggests that the hypopycnal state is enhanced, whereas brown suggests that the hyperpycnal state is reached. The numbers on the lower x-axis of Fig. 4 are the density differences between river water and seawater, simply calculated from the maximum amounts of input SSM and freshwater. If the density difference is positive (negative), the density of river water is larger (smaller) than that of seawater. From the results, there is a critical point between hypopycnal and hyperpycnal where the amount of SSM is four to six times the STD, but the state also changes depending on the particle size of SSM.

**Figure 5:**
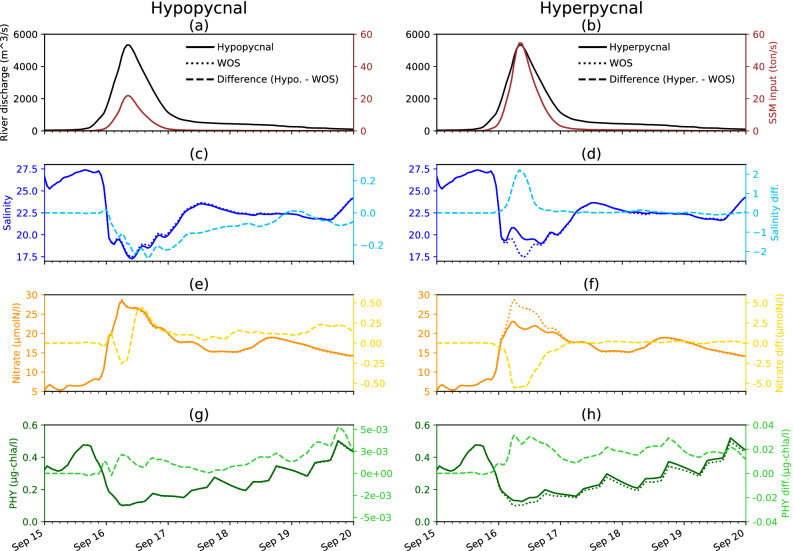
Left panels (**a**,**c**,**e**,**g**) illustrate the typical hypopycnal case (SSM input: 4, SSM diameter: 0.05 mm, nutrient input: 1 in Table 1). Right panels (**b**,**d**,**f**,**h**) show the typical hyperpycnal case (SSM input: 10, SSM diameter: 0.01 mm, nutrient input: 1 in Table 1). (**a**,**b**) Time series of river discharge (black lines; left y-axes) observed at Fukuchiyama, 37 km upstream from the mouth of Yura River, September 15–20, 2013 (Ministry of Land, Infrastructure, Transport and Tourism, Japan). Brown lines depict SSM input (right y-axes) to the river area. (**c**) Time series of salinity in the hypopycnal case (blue solid line; left y-axis), the WOS (blue dotted line; left y-axis), and the difference between the hypopycnal case and WOS (light blue dashed line; right y-axis) calculated in the surface (the top two layers) and river plume area (salinity ≤ 28.0) mean. (**d**) Time series of salinity in the hyperpycnal case (blue solid line; left y-axis), the WOS (blue dotted line; left y-axis), and the difference between the hyperpycnal case and WOS (light blue dashed line; right y-axis). (**e**,**f**) As (**c**,**d**), but for nutrient concentrations (yellow solid and dotted lines; left y-axes) and differences (light yellow dashed lines; right y-axes). (**g**,**h**) As (**c**,**d**), but for phytoplankton concentrations (green solid and dotted lines; left y-axes) and differences (light green dashed lines; right y-axes). Note that the scales of the right y-axes in (**c**,**d**), (**e**,**f**), and (**g**,**h**) are different by approximately one order of magnitude.

**Figure 6:**
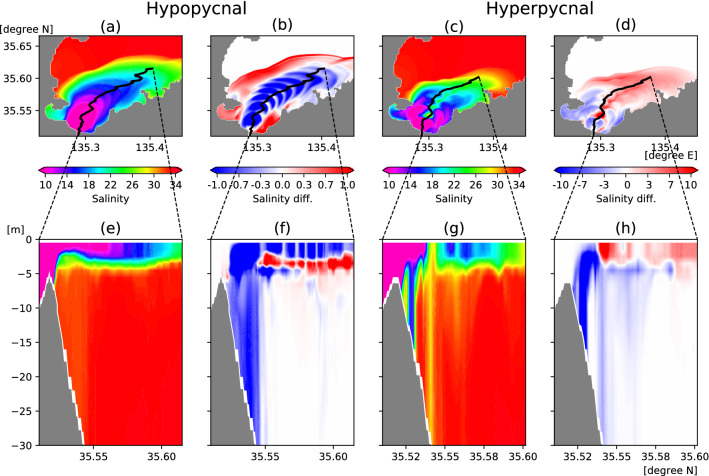
(**a**) Surface (the top two layers) mean distributions of salinity in the typical hypopycnal plume case at 16:00 LT on September 16. (**b**) As (**a**), but for the salinity difference from WOS. (**c**) As (**a**), but for the typical hyperpycnal plume case at 12:00 LT on September 16. (**d**) As (**c**), but for the salinity difference from WOS. (**e**) Vertical salinity distribution along the center section of the river plume indicated by the black line on (**a**). The black line is the main streamline of the riverine output flows determined by the SSM concentration, the flow speed, and the sea surface height maxima. (**f**) As (**e**), but for the salinity difference from WOS. (**g**) As (**e**), but for the typical hyperpycnal plume case. (**h**) As (**g**), but for the salinity difference from WOS. Note that the blue–red color scale of salinity differences differs by one order of magnitude in the hypopycnal and hyperpycnal cases. This figure was prepared with matplotlib ver. 3.3.1 in Python ver. 3.7.6.

**Figure 7:**
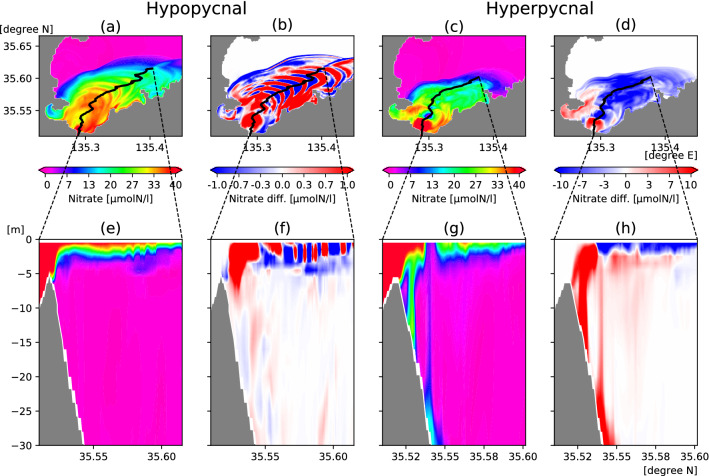
As Fig. 6, but for nutrients (nitrate). Blue–red color contrasts are opposite to those of salinity differences in Fig. 6. This figure was prepared with matplotlib ver. 3.3.1 in Python ver. 3.7.6.

**Figure 8:**
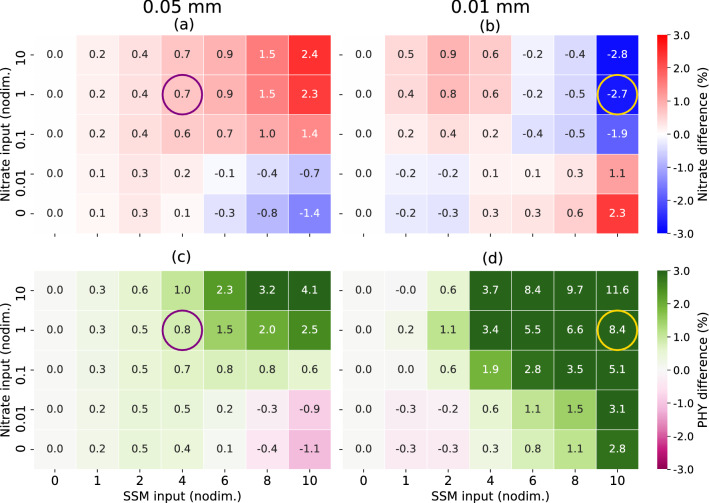
(**a**,**b**) Nutrients (nitrate) and (**c**,**d**) PHY (phytoplankton) differences from WOS in the surface (the top two layers) and river plume area (salinity ≤ 28.0) mean, in sensitivity experiments considering changes in SSM inputs (x-axes) and nitrate inputs (y-axes), when the river plume is sufficiently large (Sep. 17, 00:00 LT). Values of the x- and y-axes are normalized by those of STD. (**a**,**c**) Relatively large SSM diameter (0.05 mm) is likely to generate a hypopycnal situation. (**b**,**d**) Relatively small SSM diameter (0.01 mm) is likely to cause a hyperpycnal situation. Purple circles mark typical hypopycnal cases, yellow circles typical hyperpycnal cases.

The results demonstrate that the greater the amount of SSM and the larger the particle size (i.e., the shorter the SSM residence time), the more the hypopycnal state is enhanced (darker blue). However, there is a limit to the hypopycnal state because the transition to the hyperpycnal state (brown color) is more likely to occur when the amount of SSM is extremely large and/or the particle size is small. Because a hyperpycnal plume occurs no matter how great the amount of SSM, the high-salinity effect of a hyperpycnal state is more quantitatively influential than the lower-salinity hypopycnal state. Below we examine the temporal transitions (Fig. 5) and spatial distributions (Figs. 6, 7) of the two cases in which a typical hypopycnal process (purple circle in Fig. 4) and a typical hyperpycnal process (yellow circle in Fig. 4) occur. In this analysis, the riverine nutrient inputs are the same as in the STD, but we also demonstrate the results of a sensitivity experiment with different nutrient concentrations (Fig. 8).

The surface salinity difference is negative in a typical hypopycnal state (light blue dashed line in Fig. 5c), and is positive in a typical hyperpycnal state (light blue dashed line in Fig. 5d). The maximum salinity decrease caused by the hypopycnal process occurs 8 h after the flood peak (Sep. 16 08:00 LT in Fig. 5a), whereas the peak of salinity increase resulting from the hyperpycnal process occurs at approximately the same time as the flood peak (Sep. 16 08:00 LT in Fig. 5b). This difference arises because the hypopycnal process, in which the vertical water exchange weakens, takes longer than the hyperpycnal process, in which the river water sinks directly to the lower layer. The lower-salinity effect caused by the hypopycnal process persists for longer, but the salinity difference in both hypopycnal and hyperpycnal cases approaches zero within a few days after the flood peak (i.e., the SSM–physical interaction effect lasts only a few days). As can be seen from the right y-axes of Fig. 5c–h, the differences between the hypopycnal and the hyperpycnal cases are approximately one order of magnitude; i.e., the maximum effect of the hyperpycnal process is quantitatively stronger than that of the hypopycnal process. The positive and negative tendencies of the salinity differences (Fig. 5c,d) and the nutrient differences (Fig. 5e,f) are reversed because the concentration balances of salinity and nutrients between river water and sub-surface seawater are opposite in the STD case.

The tendency in PHY differences (light green lines in Fig. 5g,h) differs from those for salinity and nutrients. The differences tend to be positive both in the hypopycnal and hyperpycnal cases, although the difference is approximately one order of magnitude larger in the hyperpycnal. In the hypopycnal case, the increased nutrient in the surface plume is consumed by PHY, resulting in a slightly higher PHY concentration. In contrast, in the hyperpycnal case, the effect of mixing with the sub-surface chlorophyll maxima is more pronounced than the effect of the reduced surface nutrient concentration. In the short term, a large PHY difference is unlikely to occur because formation of a bloom by proliferation of phytoplankton in the surface river plume requires 5 days or more. The absolute value of PHY concentration in the surface layers (dark green solid lines in Fig. 5g,h) increases gradually with time, but the difference in concentration decreases with time. (At first glance, the difference does not seem to be smaller with time, but this is due to the fact that PHY reacts more slowly than salinity and nutrients. We have confirmed that the difference approaches zero at longer times [data not illustrated].) Due to this time lag, the maximum effect of SSM–physical interaction on the increase in surface PHY concentration is not as large as that of nutrient concentration.

After Sep. 18, the absolute values and differences of salinity, nutrient, and phytoplankton concentrations appear to fluctuate irregularly. These fluctuations result from the noise caused by expansion of the river plume beyond the model domain and inflow of seawater from outside the domain by wind, which is beyond the focus of this study. In fact, these oscillations do not occur in a simulation with a larger domain and no wind, and the difference from the SSM–physical interaction simply decays (not illustrated).

Overall, these results (Fig. 5) demonstrate that the SSM–physical interaction in the hypopycnal process decreases (increases) the surface salinity (nutrient concentration). In contrast, the hyperpycnal case exhibits the opposite tendencies. The effect is largest around the time of maximum SSM input within a lag of zero to several hours, and the effect decreases with time. The simulation run time in the present study (9 days) covers the time scale of flooding-induced PHY increase prior to a bloom occurring. However, the PHY difference caused by the SSM–physical interaction is not large over both the short and long term.

Typical spatial salinity distributions for the hypopycnal and hyperpycnal situations are illustrated in Fig. 6. The horizontal distributions show the characteristics of the hypopycnal (Fig. 6a) and hyperpycnal (Fig. 6c) processes, in which the salinity in the surface layer of the river plume becomes lower and higher, respectively. Interestingly, the riverine low-salinity water sinks partially near the flow axis in the hyperpycnal case (black line in Fig. 6c). Rather than the entire river plume sinking, the low-salinity water sinks as if a hole is made at the bottom of the thin river plume lens, and part of this water reaches the seafloor (Fig. 6g).

The patterns in the hypopycnal and the hyperpycnal cases are more obvious when comparing with the case without SSM. The hypopycnal case has lower salinity (blue color in Fig. 6b) and the hyperpycnal case has higher salinity (red color in Fig. 6d) in the surface, particularly near the flow axes. In the hyperpycnal case, the salinity also becomes higher in the offshore part of the river plume where no major sinking occurs. This result indicates that the homopycnal process (Fig. 1b) is more influential than the hyperpycnal process in the area. This combined process causes the surface layer to possess higher salinity in the hyperpycnal plume defined in this study. Due to various factors, there are also places in which the opposite patterns appear locally, but when averaged horizontally within the surface plume, the hypopycnal process decreases the salinity by up to 0.5 and the hyperpycnal process increases the salinity by up to 2.2. Vertically, at depths shallower than 5 m (Fig. 6f,h), the red–blue contrast between the surface and sub-surface layers is also reversed in the hypopycnal and hyperpycnal situations, although the color-bar scale of the differences differs by one order of magnitude. Thus, the quantitative effect is likely to be larger in the hyperpycnal situation.

For nutrient concentration, the red–blue contrast of nutrient distribution in the river plume (Fig. 7) exhibits the inverse pattern of that for salinity, because river water contains high nutrient concentrations, unlike salinity. However, this tendency depends on the balance between the high and low nutrient concentrations in the river water and the surrounding seawater. As this balance can vary considerably among various regions, we conduct sensitivity experiments in which the riverine nutrient concentrations vary from 0 to 10 times the STD. This experiment is intended to assess situations in which river water nutrients are lower to higher than in the marine sub-surface layer. The relationships of nutrients and PHY in the river plumes between the amount of riverine SSM (x-axes) and the riverine nutrient input (y-axes) are plotted as heat maps (Fig. 8) for both large SSM particle size (0.05 mm), with which a hypopycnal process is likely to occur (Fig. 8a,c), and small particle size (0.01 mm), which is likely to generate a hyperpycnal situation (Fig. 8b,d). There is a critical value of approximately 0.01 to 0.1 on the vertical axis, above which the riverine nutrient concentration (i.e., surface nutrient concentration) is higher than the sub-surface concentration, and below which the riverine nutrient concentration is lower. The further to the right on the horizontal axis, the larger are the absolute values of the differences resulting from the SSM–physical interaction. The interactive processes produce consistent results for the hypopycnal and hyperpycnal cases. In the hypopycnal case (Fig. 8a), the surface water tends to become more isolated, so the surface layer becomes more nutrient-rich (poor) when the riverine nutrient concentration is higher (lower) than the sub-surface layer. In the hyperpycnal case (Fig. 8b), the opposite tendency is observed because the surface water and sub-surface water tend to mix. Thus, the SSM–physical interaction can have opposite effects on river-originated tracers, such as nutrients, depending on the surrounding situation. The larger (smaller) the difference in concentration between river water and the sea sub-surface, the larger (smaller) is the quantitative impact.

The effect of consumption by PHY is not yet apparent in the nutrient concentration differences, because the phytoplankton are still consuming the nutrients. In the hypopycnal case (Fig. 8c), the trend is similar to that of nutrient concentration difference, but the trends differ in the hyperpycnal case (Fig. 8d). This result reflects the mixing effect with water in the lower layer, which contains a relatively higher PHY concentration due to the sub-surface maxima, rather than the effect of increasing or decreasing surface nutrients.

## Discussion and conclusions

To elucidate the impact of SSM–physical interaction on nutrient circulation and primary productivity during flooding river discharge, we conducted numerical simulations for a specific flooding event of the Yura River. The amount and particle size of SSM, for which there are large uncertainties at flooding times, were varied over wide but realistic ranges. Two qualitatively different regimes of riverine plumes appeared within these parameter ranges: hypopycnal and hyperpycnal processes. Hyperpycnal processes tend to occur with a greater amount of SSM and/or a smaller SSM particle size. Compared with the reference case in which the effect of SSM is neglected, the surface salinity within the riverine plume becomes lower in hypopycnal cases and higher in hyperpycnal cases within a few days after the flooding discharge. The salinity varies up to 12.3% on average in the surface river plume within the parameter ranges. In contrast, the nutrient content of the surface water becomes slightly higher in hypopycnal cases and markedly lower in hyperpycnal cases. In hyperpycnal cases, however, the buoyant motion of the plume after settling of the SSM particles causes the nutrient content of the subsurface water to be notably higher than in the reference case. The associated nutrient concentration in the surface river plume can vary by up to 36.8%, depending on the balance between nutrient concentrations in the river and the sea sub-surface.

The actual evolution of hyperpycnal plumes is deeply associated with the resuspension of SSM from the seafloor^21, 22^. This process is yet to be implemented in our model, and the effect of hyperpycnal plumes is likely to be underestimated in our results. The effects of photosynthesis inhibition caused by shading of sunlight by SSM^23^ are not also employed in the simulations. The light-attenuation effect often delays and smooths the onset of a phytoplankton bloom^10^. It has been also reported that high turbidity water near a river mouth moved high concentration chlorophyll areas from the coast to offshore^24^. However, the SSM–physical interaction here is mainly effective several days after the flood peak when the phytoplankton bloom is not fully activated; therefore, even if the effect is introduced into our model (i.e., light limitation delays the plankton bloom and shifts the high chlorophyll region seaward to some extent), our results would not change fundamentally in terms of SSM–physical interaction. This is not the case if the aforementioned resuspension process is introduced and a hyperpycnal plume persists for more than a few days. Such extraordinary cases will be a future challenge.

We emphasize that qualitatively different situations for the supply of nutrients to the surface and subsurface waters are generated within realistic ranges of parameters for the SSM properties. Although our calculation is only for a specific event of a specific river, our results are applicable to other flooding events and other rivers and connecting coastal seas. Therefore, it is important to properly take into account the effect of SSM in assessing the influence of high river discharges on the biogeochemistry of coastal seas. The intensity and frequency of extreme events, including flooding, are considered to become higher under a warming climate^25^, which would also increase the impact of SSM in riverine discharges on coastal seas.

## Methods

### Model description

The coastal sea of focus here is an open bay facing the open ocean (Fig. 2). The width and length of the bay (~ 18 and ~ 21 km, respectively) are on a scale at which the effect of Earth's rotation is influential. The bay has simple surrounding conditions: only one river (the Yura River) dominates the freshwater supply to the bay, and tidal amplitudes are substantially small around the Sea of Japan. The sum of the amplitudes of four major tidal components (M2 + S2 + K1 + O1) is estimated to be 19 cm at most in the Tango Bay^26^. It is reasonable to neglect their effect, particularly during flood events. From available observed data^19^, the largest flood experienced by the Yura River occurred in Sep. 2013.

The flood is simulated by a non-hydrostatic ocean model^27^. The detailed physical and SSM simulation settings follow the realistic setting of HO19^4^. The only differences from HO19 are the change in the vertical resolution of the top layer from 5 to 1 m, and the associated initial values of temperature and salinity, resulting in the model simulation being closer to reality. The initial condition and distributions are closer to observations (Supplementary Figs. S1, S2).

We also introduce a simple lower-trophic level ecosystem model^28^ (OY05 hereafter) into HO19. OY05 is parameter-tuned for the Japan Sea, which is suitable for the bay facing the Japan Sea of the present study. Most of the parameter values used here are the same as OY05, but the optimum light intensity is changed from 70 to 65 W/m^2^ to be closer to the observed phytoplankton distribution in the bay (Supplementary Fig. S2). This ecosystem model runs on a nitrogen basis, which is reasonable because nitrogen is often the main limiting factor for phytoplankton growth compared to phosphorus, silicon, and iron in the bay in summer^29^.

The initial conditions of the ecosystem part (Supplementary Figs. S1, S2) are created as follows. The physical condition from September 11 to 14, 2013, before the flood is repeated and the mean Yura River’s discharge in 2013 (62.46 m^3^/s) is poured. The ecosystem model is run by the physical field for 60 days, and the resulting distributions are used as the initial condition. The nutrient concentration from the river is set to 0.046 mol/m^3^ as STD (standard case), referred to in a previous study^29^. The light intensity is adopted from the shortwave radiation 3-hourly data of JRA55-do^30^. The light intensity distribution is spline-interpolated into the model grid and converted into photosynthetically active radiation. The same nutrient concentration and light intensity are used as the boundary condition of the simulations after the targeted flood.

The initial distributions thus produced are compared with satellite data and field observations. The satellite data were collected by a geostationary satellite, Communication, Ocean and Meteorological Satellite (COMS) carrying an ocean color sensor, Geostationary Ocean Color Imager (GOCI). The satellite-observed distributions of sea surface salinity and chlorophyll concentration in Tango Bay on Sep. 11, 2013, before the targeted flood were estimated by the algorithms for salinity^31^ and chlorophyll concentration^32^. The in situ data were measured by shipboard observations in Tango Bay also on Sep. 11, 2013. Vertical temperature, salinity, and chlorophyll concentration were based on surveys using a Conductivity-Temperature-Depth (CTD) profiler (Alec Compact-CTD, JFE Advantech). The modeled horizontal salinity distribution before the flood (Supplementary Fig. S1b) is in good agreement with the satellite-estimated and the field observation data (Supplementary Fig. S1a). For chlorophyll concentrations, the satellite data are an overestimate (Supplementary Fig. S1c,d), although the simulation result and the in situ data are close. Coastal chlorophyll concentrations obtained by satellite tend to be overestimated compared to those in the open ocean^33, 34^. The vertical distributions of the simulations at stations A and B (Supplementary Fig. S2) also reproduce the field observations to some extent. There are no in situ data for nitrate, but the results are somewhat consistent with the values observed in 2010 and 2011^29^; thus, this simulation reproduces the bay reasonably well, at least prior to the targeted flood. During the flood, there are no in situ data, and satellite observations cannot estimate salinity below 22.0 due to the algorithm. Chlorophyll concentrations obtained by satellite also tend to be overestimated in coastal regions. Therefore, field and satellite observations are not compared with the simulation results during the targeted flood in this study. As such, numerical modeling is an important tool, especially during floods.

### Sensitivity experiments and analysis

In HO19, sensitivity experiments were conducted, with a range of values of the parameters of SSM flux into the river, SSM composition, and SSM-particle diameter. Of the three parameters, the SSM–physical interaction effects on the surface freshwater volume were most pronounced when the amount of SSM and the SSM particle size were changed; therefore, the sensitivity experiments of the present study involved changing the amount and particle size of SSM. In addition, sensitivity experiments with alternations in the riverine nutrient concentration are conducted to investigate the effects on the ecosystem (Table 1). The parameter values of the standard case (STD) are estimated from empirical equations^35^ and past studies^29^. The values of SSM amount and nutrient concentration are set to between zero and ten times the STD, yielding a total of 155 cases. We here focus on the SSM–physical interaction, so we discuss the difference between the reference cases in which the effect of SSM is neglected (WOS) and the other SSM-effective cases. In the SSM-effective cases, the phenomena shown in Fig. 1 often occur in a complex manner. The case in which the process shown in Fig. 1a appears most clearly in the spatial distribution is defined as the typical hypopycnal case, while the case in which the process shown in Fig. 1c appears most strongly is defined as the typical hyperpycnal case.

In defining the river plume caused by the flood, the thickness of the surface layer is defined as ~ 2 m (upper two layers in the model), and the area below salinity 28 in the STD case is defined as within the river plume. These values are almost the same as the definition used in HO19 for the flood simulation. The only difference is that the surface layer is defined to be ~ 2 m (compared with ~ 5 m in HO19); this difference arises because the model used in the present study has a finer vertical resolution than the previous model.

## Supplementary Information

Supplementary Information 1.

Supplementary Information 2.

## Data Availability

River discharge data of the Yura River in September 2013 were obtained from the Water Information System of the Ministry of Land, Infrastructure, Transport and Tourism (MLIT), Japan (http://www1.river.go.jp/cgi-bin/DspWaterData.exe?KIND=6&ID=306091286605030&BGNDATE=20130901&ENDDATE=20191231&KAWABOU=NO). The remote sensing products were downloaded from the website (http://kosc.kiost.ac.kr/index.nm) of the Korea Ocean Satellite Center (KOSC). Coastline data for Tango Bay were downloaded from the website (https://nlftp.mlit.go.jp/ksj/) of the National Land Information Division, National Spatial Planning and Regional Policy Bureau, MLIT, Japan. The files necessary to reproduce the simulations and the in situ data are available from the authors upon request. The model used in this study (kinaco) is available online at http://lmr.aori.u-tokyo.ac.jp/feog/ymatsu/kinaco.git/.
