# Supplementary material for: Biogeochemical impacts of flooding discharge with high suspended sediment on coastal seas: a modeling study for a microtidal open bay
Source: Sci Rep. 2021 Nov 4;11:21322. doi: 10.1038/s41598-021-00633-8 (PMC8568930; doi:10.1038/s41598-021-00633-8)
Supplement: Supplementary file 1 — Supplementary Information 1. [file 41598_2021_633_MOESM1_ESM.docx]

Supplementary Figure Captions

Supplementary Figure S1. (a) Surface salinity at 1 m depth in Tango Bay on Sep. 11, 2013, before the targeted flood. Data were obtained from satellite (contour) and in situ (dots) observations. (b) As (a), but for the initial state of the model simulations (the top layer). (c) As (a), but for phytoplankton concentration. (d) As (b), but for phytoplankton concentration. Black circles (A, B) mark the locations of the points detailed in Supplementary Fig. S2. This figure was prepared with matplotlib ver. 3.3.1 and cartopy ver. 0.17.0 in Python ver. 3.7.6. We also used the coastline data from the National Land Information Division, National Spatial Planning and Regional Policy Bureau, MLIT of Japan (https://nlftp.mlit.go.jp/ksj/gml/datalist/KsjTmplt-C23.html).

Supplementary Figure S2. Vertical temperature, salinity, nitrate, and phytoplankton distributions measured on Sep. 11, 2013 at Point A and Point B of Supplementary Fig. S1.
