# Supplementary figures and images for: Biogeochemical impacts of flooding discharge with high suspended sediment on coastal seas: a modeling study for a microtidal open bay
Source: Sci Rep. 2021 Nov 4;11:21322. doi: 10.1038/s41598-021-00633-8 (PMC8568930; doi:10.1038/s41598-021-00633-8)

**Fig. S1**

Satellite & in situ  
(a)

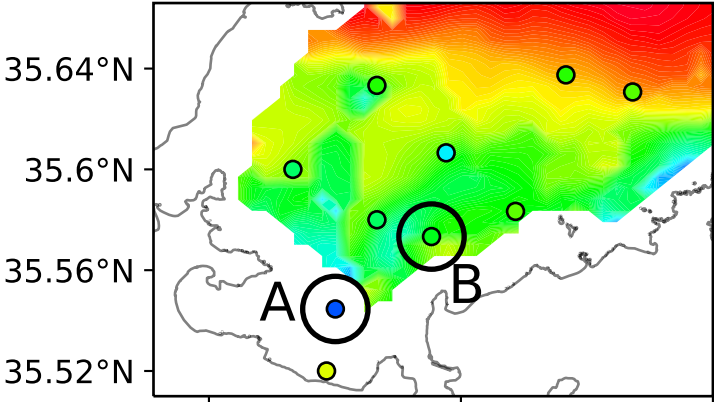

Model  
(b)

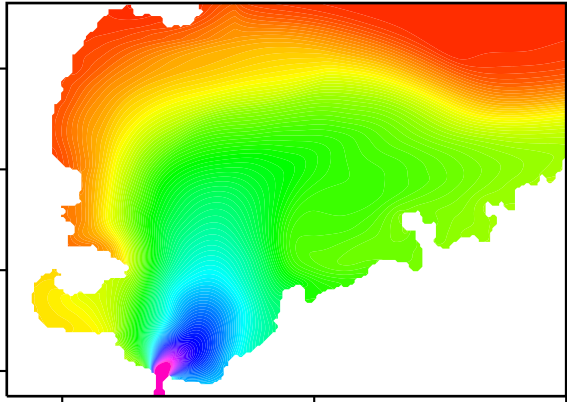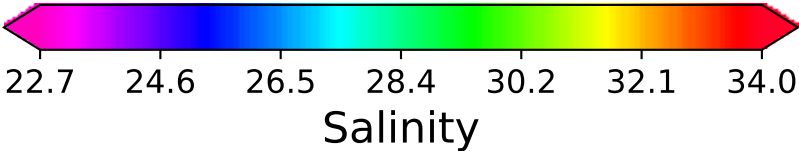

(c)

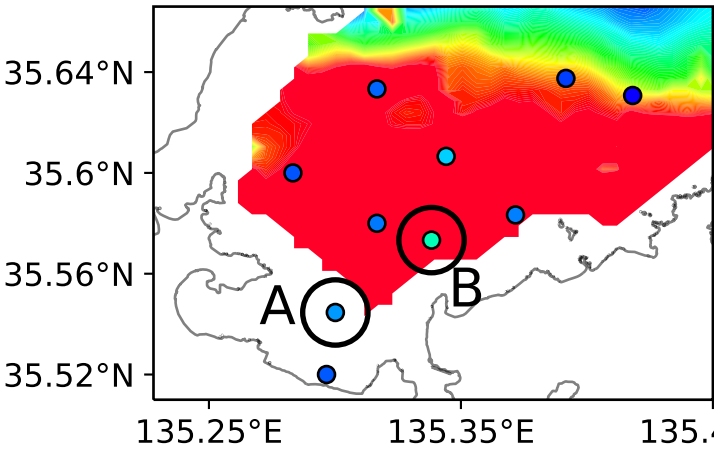

(d)

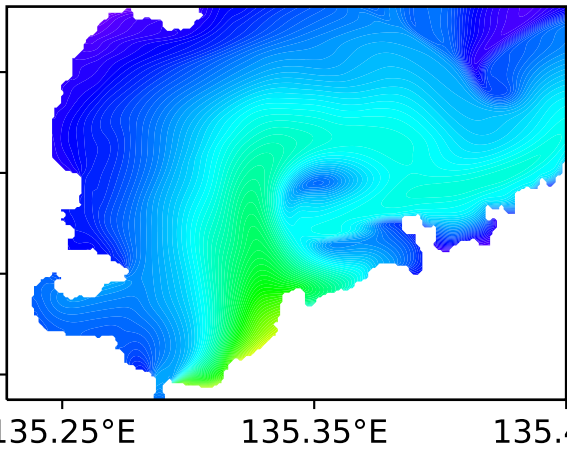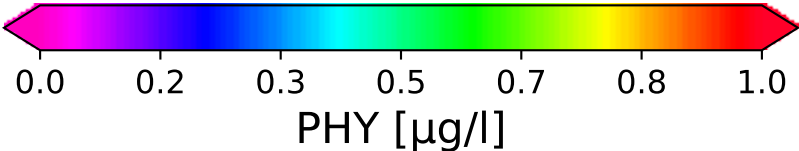

**Fig. S2**

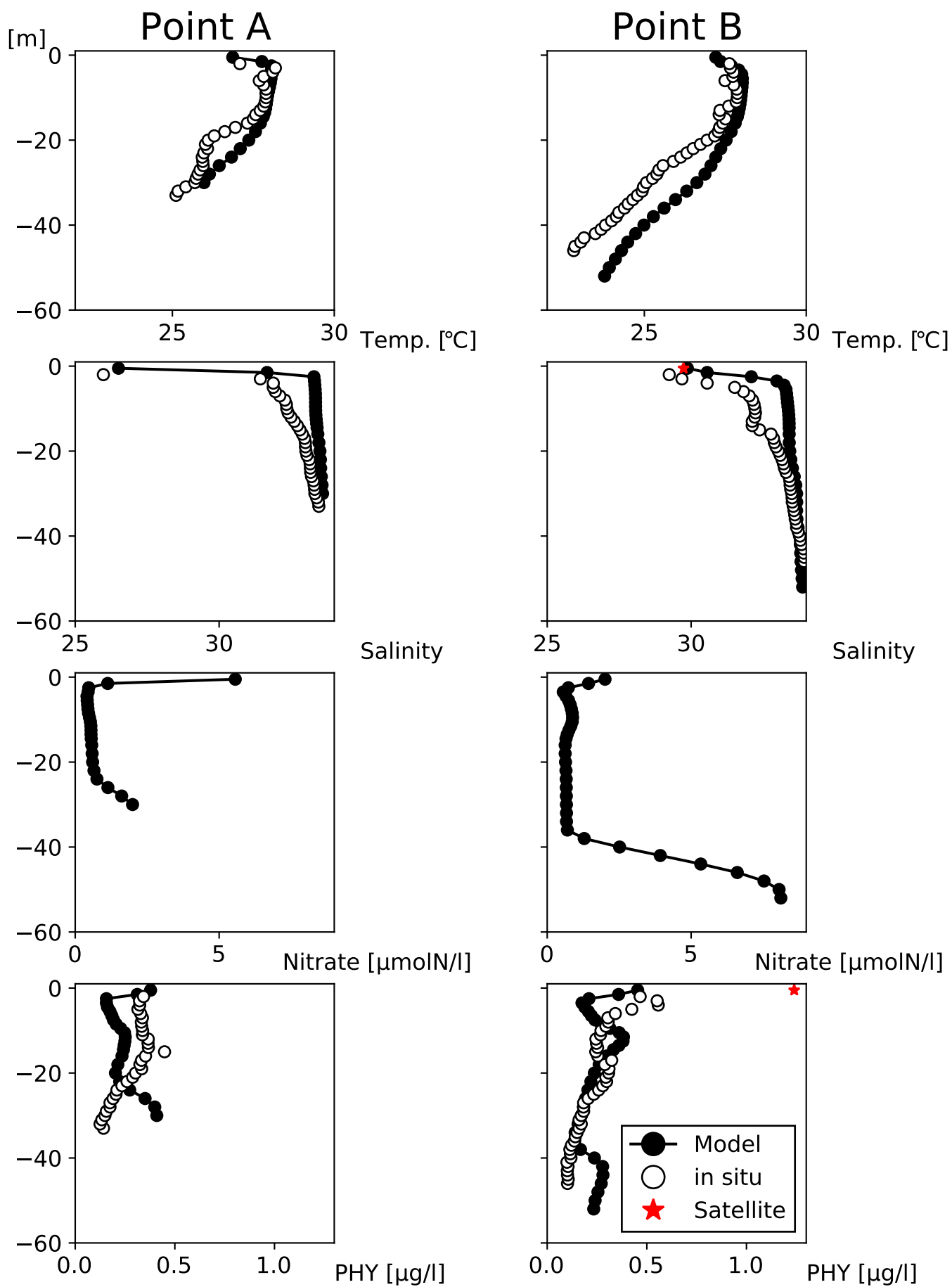

Supplement: Supplementary file 2 — Supplementary Information 2. [file 41598_2021_633_MOESM2_ESM.pdf]
